# Successful immunization of naturally reared pigs against porcine cysticercosis with a recombinant oncosphere antigen vaccine

**DOI:** 10.1016/j.vetpar.2012.03.055

**Published:** 2012-09-10

**Authors:** César M. Jayashi, Craig T. Kyngdon, Charles G. Gauci, Armando E. Gonzalez, Marshall W. Lightowlers

**Affiliations:** a Veterinary Clinical Centre, The University of Melbourne, 250 Princes Hwy, Werribee, Victoria 3030, Australia; b Faculty of Veterinary Medicine, Universidad Nacional Mayor de San Marcos, Avenida Circunvalación cda 28 s/n San Borja, Lima, Peru

**Keywords:** Neurocysticercosis, Cysticercosis, Taeniasis, TSOL16, TSOL18, Vaccine, Immunization, Pigs, Field trial

## Abstract

*Taenia solium* causes cysticercosis in pigs and taeniasis and neurocysticercosis in humans. Oncosphere antigens have proven to be effective as vaccines to protect pigs against an experimental infection with *T. solium.* A pair-matched vaccination trial field, using a combination of two recombinant antigens, TSOL16 and TSOL18, was undertaken in rural villages of Peru to evaluate the efficacy of this vaccine under natural conditions. Pairs of pigs (*n* = 137) comprising one vaccinated and one control animal, were allocated to local villagers. Animals received two vaccinations with 200 μg of each of TSOL16 and TSOL18, plus 5 mg Quil-A. Necropsies were performed 7 months after the animals were distributed to the farmers. Vaccination reduced 99.7% and 99.9% (*p* < 0.01) the total number of cysts and the number of viable cysts, respectively. Immunization with the TSOL16–TSOL18 vaccines has the potential to control *T. solium* transmission in areas where the disease is endemic, reducing the source for tapeworm infections in humans.

## 1 Introduction

*Taenia solium* is a taeniid cestode parasite which causes taeniasis in humans and cysticercosis in humans and pigs. When the cysticerci affect the central nervous system the disease is called neurocysticercosis. Neurocysticercosis is a zoonotic disease which is widespread in the developing world. In Latin America there are more than 400,000 symptomatic neurocysticercosis cases with almost 10% of these cases occurring in Peru (Bern et al., 1999). The life cycle of *T. solium* involves both humans and pigs. The adult tapeworm lives in the human small intestine. Tapeworm eggs are shed in the feces and if ingested by a pig, the larvae or cysticerci develop principally in the muscle of the pig. Humans become infected with the adult tapeworm after ingesting raw or incompletely cooked pork meat infected with the larval stage (cysticercus). However, the pathological consequences of *T. solium* infection in humans arise because humans may also become accidental intermediate hosts because of ingestion of tapeworm eggs. In humans the eggs develop into larval cysts that are mainly located in the muscular and nervous tissues. The clinical significance of the parasite occurs particularly when cysts that are located in the central nervous system cause serious neurological disorders such as seizures and epilepsy (Schantz et al., 1993; Garcia and Del Brutto, 2005).

Logically, *T. solium* control could be achieved by improvements in public sanitation (through health education), treatment of humans to remove the adult tapeworms, preventing pigs having access to human faces, inspection of pork meat to prevent infected material being available for consumption, or by killing the cysticerci in the intermediate host with anthelmintics (Garcia and Del Brutto, 2005; Gonzalez et al., 2001; Pawlowski et al., 2005; Sarti et al., 2000). Despite the availability of these potentially effective methods to interrupt the parasite's life cycle, cysticercosis and neurocysticercosis remain serious problems in many developing countries (Garcia and Del Brutto, 2005).

A potential method for controlling *T. solium* transmission is the use of vaccination in pigs (Cai et al., 2008; Flisser et al., 2004; Gonzalez et al., 2005; Lightowlers, 1999, 2010; Sciutto et al., 1990). Using an effective vaccine in pigs would remove the source of tapeworm infection in humans, breaking the parasite's life cycle and indirectly eliminating the causative agent of human neurocysticercosis (Lightowlers, 1999, 2010). Oncosphere antigens have been found to be highly effective as vaccines for prevention of infections with parasites that are closely related to *T. solium* such as *Taenia ovis* (Johnson et al., 1989), *Taenia saginata* (Lightowlers et al., 1996b) and *Echinococcus granulosus* (Lightowlers et al., 1996a). *T. solium* homologs of protective antigens for the other taneiid species have been identified and the recombinant proteins expressed in *Escherichia coli.* These antigens (TSOL16, TSOL18 and TSOL45) have been produced as glutathione-S-transferase (GST) fusion proteins. TSOL18 and TSOL45 have shown high efficacy as vaccines in preventing pig infections with *T. solium* in experimental trials in Mexico, Cameroon, Peru and Honduras (Flisser et al., 2004; Gonzalez et al., 2005; Lightowlers, 2006). In a recent vaccination study, the use of TSOL18 plus oxfendazole prevented any detectable infection with *T. solium* in pigs raised under natural conditions (Assana et al., 2010). The TSOL18 gene is highly conserved in isolates of *T. solium* from various geographical locations of the world (Gauci et al., 2006). However, concerns remain about how genetic variability may affect the effectiveness of the vaccine in control programs. The third oncosphere antigen, TSOL16, has also been demonstrated to be effective against experimental challenge with *T. solium* eggs (Gauci et al., in press). The availability of multiple protective antigens allowed the possibility of utilizing more than one antigen in a *T. solium* vaccine, potentially reducing the likelihood of selection for resistant genotypes during practical application of the vaccine. Here we provide data obtained from a field trial using a combination of the TSOL16 and TSOL18 antigens in naturally reared pigs in a region of Peru where *T. solium* cysticercosis is endemic.

## 2 Materials and methods

### 2.1 Study design

This was a pair-matched vaccination trial to assess the effectiveness of vaccination to prevent porcine cysticercosis infection in naturally reared pigs.

### 2.2 Animals

The number of animals required for the field trial was calculated using Fisher's exact test, taking into consideration the prevalence of porcine cysticercosis in the study area being 10%; a 90% reduction in the occurrence of the disease in the pigs vaccinated with the TSOL16–TSOL18 vaccine; a power of 80% and a significance level of 95%. On this basis, a minimum of 100 pairs of pigs were required for the study. The number of animals used in the study exceeded this minimum requirement, with a total of 274 animals being delivered in pairs to 86 households. Piglets ranging from 8 to 16 weeks were sourced from farms within the department of Piura, Peru. Pigs were “criollo” pigs or “criollo” crosses with several pig breeds (Landrace, Hampshire, Duroc, Poland China, Pietrain). Tongue inspection was performed as a screening test to avoid purchasing infected animals. None of the examined animals were positive to tongue inspection.

### 2.3 Study area

Morropon is one of the 10 districts of the province of Morropon, Piura, Peru. It is located in Northeast Peru, 82 km from Piura, the closest major city. It has a population of 16,510 inhabitants. The altitude is 131 m above sea level and geographic coordinates are 5°10′57″ south latitude and 79°58′00″ west longitude. The climate is dry and hot during most of the year. Commonly, pigs are raised free roaming and if not, very rudimentary pig-pens are used to enclose them.

### 2.4 Recombinant antigen and vaccine preparation

The cDNA of TSOL16 (Gauci and Lightowlers, 2003) and TSOL18 (Gauci et al., 1998) were cloned into pGE (GE Healthcare), and expressed as GST fusion proteins in *E. coli*, as described by Flisser et al*.* (2004) and Gauci et al. (in press). One dose of vaccine consisted of 200 μg each of TSOL16-GST and TSOL18-GST plus 5 mg Quil-A (Brenntag Biosector, Frederikssund, Denmark). The TSOL16 and TSOL18 purified protein elutions were sterilized by filtration through a 0.22 μm membrane filter prior to use. Quil-A solution was prepared in PBS and sterilized by filtration through a 0.22 μm membrane filter. Quil-A was added to an equivalent of 5 mg per dose, Tris buffer at 50 mM was added to 100% final volume and the vaccine lyophilized and stored at 4–10 °C in vials of 10 and 25 doses. Vaccine was rehydrated immediately before use using sterile distilled water.

### 2.5 Vaccination scheme

Animals were randomly assigned to the treatment (vaccinated) and control groups. Piglets in the treatment group were vaccinated intramuscularly in the neck with a dose of the TSOL16–TSOL18 vaccine per animal and Classical Swine Fever (CSF) vaccine (Pest-Vac, Fort Dodge-Wyath) at day 1, in separate injection sites. Identical booster vaccinations with TSOL16–TSOL18 and the CSF vaccines into their same respective vaccine sites, occurred four weeks after the first vaccination.

Piglets in the control group were vaccinated intramuscularly only with the CSF vaccine and given an identical booster vaccination four weeks after first vaccination. To reduce the likelihood of a CSF outbreak in our study houses, all other pigs from the household were vaccinated against CSF.

### 2.6 Management of the animals

All pigs were purchased, acclimatized for a period of 48 h and then vaccinated according to the vaccination scheme. This period was used to monitor for the appearance of any detrimental health condition and treat it before giving the pigs to their new owners. Piglets were distributed to the recipient households as pairs of sentinel pigs, consisting of one vaccinated and one control animal. Animals were bought and delivered in four lots, delivering the first group in January–February 2009 (74 animals), second in March 2009 (42 animals), third in June 2009 (58 animals) and the last group in October 2009 (100 animals).

### 2.7 Household selection criteria and delivery

Properties where the trial animals were to be hosted were selected following various criteria. In the first criterion, selected properties where at least one pig which scored strongly positive (to ≥4 bands) in the Electro-Immunotransfer-Blot (EITB) assay for porcine cysticercosis. These data had been determined three months earlier during a sero-epidemiological survey of the villages. The second criterion was to deliver piglets to neighboring households within a 50-m radius from the households at which a strongly serologically positive pig had been detected. The finding of a strongly serologically positive pig for *T. solium* cysticercosis would suggest the likelihood of *T. solium* transmission either at this property or in the immediate vicinity (Lescano et al., 2007). The household owners did not know the animals belonged to any specific treatment group. To avoid any alteration of the natural feeding behavior of pig in the field, owners were instructed to raise the pigs according to their usual practices.

### 2.8 Transport to a cysticercosis low-risk area

After the animals had remained at their host properties for approximately 4 months, the animals were transported to a facility that was considered to be a very low risk for further exposure of the animals to *T. solium*. This facility was the Cysticercosis Working Group in Peru (CWGP) rural campus located in Tumbes, Peru. Animals stayed in pens located inside the campus for 10–12 weeks providing sufficient time to allow any *T. solium* infections that had been acquired by the animals immediately before the completion of the field exposure period, enough time to develop into detectable cysticerci.

### 2.9 Necropsy

Necropsies were performed 7 months after the first vaccination to determine the incidence of the disease and intensity of infection. The viability of cysticerci was assessed on the infected animals. Personnel carrying out the necropsies and cyst counting did not know the group to which the individual animals belonged. Animals were euthanized following the ethics policies and proceedings authorized by the Universidad Nacional Mayor de San Marcos, Lima, Peru. Muscles were sliced with sagittal cuts, endeavoring not to exceed 3 mm between cuts. Cysts were classified into healthy and degenerated cysts. Healthy cysts were translucent vesicles filled with transparent fluid of variable size from 5 to 15 mm in diameter with an oval or round shape. Inside the healthy cysts a visible white scolex could be found. Degenerated cysts were small vesicles that varied from whitish to yellowish color and had a dense fluid. As a general characteristic, degenerated cysts were smaller than the healthy cyst and had the appearance similar to that of a grain of rice.

### 2.10 Statistical analysis

Comparisons between the incidence of infection in the pairs of animals from the vaccinated and control groups were calculated by the McNemar-chi^2^ test (95% significance level). Comparison of the cyst counts per group was calculated by the Wilcoxon signed-rank test (95% significance level). Fisher's exact test was used to compare the degenerated:viable cyst ratios of the control and vaccinated group. The adjusted relative risk, 95% confidence intervals and *p* values of acquiring the disease during their time of exposure were calculated using a multivariate Poisson regression. Odds ratios (OR) were calculated to compare the number of cysts in each anatomical region between the vaccinated and control groups.

## 3 Results

From the 274 animals involved in the trial, 220 were included in the necropsies. Pigs not assessed by necropsy examination either died or were lost during the trial. The 54 lost animals represented 19.7% (54/274) of the total number of pigs delivered. From the 220 euthanized pigs, 113 animals belonged to the treatment group and 107 to the control group.

A summary of the incidence, total, viable and degenerated cyst counts, as well as the viable/degenerated ratio of both groups is shown in Table 1. Numerous cysticerci were found in the 18 infected control animals (prevalence = 16.8%, total number of cysticerci = 34,081, mean = 1893.4, median = 63.5, range = 1–18,451). From the 113 animals vaccinated with the TSOL16–TSOL18 vaccine, 93.8% (106/113) pigs were free of infection. There was a significant 99.7% reduction (Wilcoxon signed-rank test, *p* < 0.01) in the total number of cysts in the vaccinated group (Total number of cysticerci = 83, mean = 11.9, median = 6, range = 1–54).

The degenerated cysts found in the controls represented 2.0% (665/34,081) of the group's total cyst count, whereas the degenerated cysts in the vaccinated group represented 96.4% (80/83) of the group's total cyst count. There was a significant 99.9% (Wilcoxon signed-rank test, *p* < 0.01) reduction in the number of viable cysts in the vaccinated group (viable cysts = 3, mean = 1.5) versus the animals in the control group (viable cysts = 33,416, mean = 2227.7).

In the control animals, for every 50 viable cyst 1 degenerated cyst was found (degenerated:viable ratio = 0.02 [665/33,416]). On the other hand, in the vaccinated group, for every viable cyst 27 degenerated cysts were found (degenerated:viable ratio = 26.67 [83/3]). These viable/degenerated ratios were significantly different (Fisher's exact test *p* < 0.001).

The number of infected pairs by group is shown in Table 2. The infected animals belonged to 20 pairs. There were 19 out of the 20 positive pairs that had their matching pair for the necropsy examination, and 17 pairs (from the 137 starting pairs) where both pigs were unavailable to assess their infection status because they either died or otherwise lost during the experiment. There was a significant difference between the incidence of infection in the treatment and control groups when analyzing the pairs of pigs (McNemar-chi^2^ test, *p* < 0.05). Using a multivariate analysis and adjusting the regression by village, house and sex, the risk of finding an animal with viable cysts and with muscle viable cysts was 11.53 (95% CI 1.73–21.34) and 12.61 (95% CI 2.79–22.45) times more in the control than the vaccinated group, respectively.

A summary of the cyst counts in the infected animals per group is given in Table 3. From seven infected animals in the vaccinated group, five had degenerated cysts and two animals had viable cysticerci. From these two animals, one had two viable cysts in the brain only and the other had one viable muscle cyst. Localization of cysts in both groups had a similar pattern, 53.0% (vaccinated) and 54.3% (control) of the cysts were localized in the limbs, while the remaining cysts found in the tongue and the heart as well as spinal, intercostal and head muscles. Animals in the control group with a parasite burden >1084 cysts, had cysts in skeletal muscle, cardiac muscle and brain parenchyma. Brain cysts were found in 38.9% (7/18) of the infected controls. The animals in the control group had statistically significantly lower odds to have cysts in the head muscles (OR = 2.73 [1.26–5.93]), tongue (OR = 8.03 [4.24–15.22]) and brain (OR = 8.94 [2.17–36.92]). A distribution of the anatomical location of cysts is shown in Table 4.

## 4 Discussion

This study reinforces the high efficacy of the oncosphere antigen vaccine against porcine cysticercosis and the potential for use of the vaccine in programs to control neurocysticercosis. The effectiveness of vaccination (>99%) reported in this trial is as high as the vaccine effectiveness found in studies that used controlled conditions (Flisser et al., 2004; Gonzalez et al., 2005). Under natural conditions the effectiveness of the TSOL16–TSOL18 vaccine for the control of porcine cysticercosis in Peru was similar to that described for use of TSOL18 as a vaccine in the experiment undertaken by Assana et al. (2010) in Cameroon. However, there are some differences between the trial in Peru and the trial undertaken in Cameroon. In Peru the effectiveness of vaccination alone was assessed without the incorporation of an oxfendazole treatment in the trial animals, as was undertaken in Cameroon. Furthermore, the Peruvian trial incorporated a second recombinant antigen in the vaccine (TSOL16), whereas in Cameroon only TSOL18 was used alone. Despite the differences, both trials achieved a >99% reduction in the total number of cysts as well as the number of viable cysts in vaccinated animals.

Animals in the control group of our study had more than 12 times the risk of having a viable cyst compared to the animals in the vaccinated group. Viable cysts are the only ones that could have developed into the adult parasite. Although the three viable cysts (two in brain and one in muscle) found in the vaccinated animals had the potential to develop into adult tapeworms, only the one viable muscle cyst would have been likely to perpetuate the life cycle because the consumption of the brain is a rare practice among the rural villagers (Gonzalez et al., 1998).

The TSOL antigens are expressed exclusively in the oncosphere (Gauci et al., 1998) and for this reason vaccination with these antigens would not be expected to have any effect on established cysticerci. Nevertheless, there is some evidence in this field vaccination trial, which suggests that protection provided by the TSOL16 and TSOL18 antigens led to an increase in the number of non-viable cysticerci in the vaccinated animals. Previous studies in *T. solium* and *Taenia taeniaeformis* have reported similar findings. Vaccination with S3Pvac vaccine (a *Taenia crassiceps*-based synthetic peptide vaccine) against *T. solium* cysticercosis, found that cysticerci were susceptible to the immune responses induced by the vaccine (de Aluja et al., 1999). The higher ratio of degenerated cysts observed in the vaccinated group of our study, resembles a pattern similar to Bøgh et al. (1988) and Rajasekariah et al. (1980) findings in *T. taeniaeformis*, with an increased number of degenerated cysts. Other evidence relating to the factors which affect the viability of a muscle lesion caused by *T. solium* in pigs is that animals infected with *T. solium* eggs and then re-infected 130 days later shown an almost 1:1 ratio of degenerated and viable cysts (de Aluja et al., 1996, 1999). Overall in our study, degenerated cysts represented 96.5% of the total cyst count in the vaccinated group versus 2% in the control group. Future research could assess the potential cysticidal effect of the TSOL16–TSOL18 vaccines.

The precise duration of immunity conferred by vaccination with TSOL antigens is unknown. In both the study detailed in this paper and the trial described by Assana et al. (2010), the vaccine was effective in achieving high levels of protection that were assessed at the times necropsies were undertaken at the conclusion of the trials. In our study in Peru, this was 7 months after the initial immunizations. In the case of the trial undertaken using TSOL18 in Cameroon, protection was maintained for at least 10 months after the first immunization (Assana et al., 2010).

This study has some limitations. Despite the precautions taken in selecting the pigs for the vaccination trial, the infection seen in the animals could not unequivocally be determined as having been acquired while the animals were at the villages of Morropon. Animals may have been infected before being distributed to the households. Indeed, serological investigations undertaken after the completion of the trial indicated that some of the animals may have been infected with *T. solium* at the time they were initially distributed to the householders. Hence, the lesions seen in the vaccinated group cannot be regarded unequivocally as vaccine failures.

### 4.1 Pig vaccination in control programs

In establishing a control program for *T. solium*, care would need to be taken in relation to the endemic stability of disease in pigs. As is known to be the situation for other taeniid cestode species (Gemmell, 1990), it could be anticipated that areas where *T. solium* cysticercosis was hyper-endemic would exhibit endemic stability with respect to infection levels in pigs. Disturbance of this stability by reduction in the prevalence of infected definitive hosts could reduce the incidence if infection in pigs, leaving many highly susceptible to any future exposure to the parasite. This situation is believed to have led to the occurrence of “cysticercosis storms” in New Zealand where disturbance of endemic stability of *T. ovis* in sheep led to massive infections in flocks that were exposed to *T. ovis* eggs (Gemmell, 1978) and is a well understood phenomenon in relation to other infectious diseases (Coleman et al., 2001). Most of the interventions that have been tested for *T. solium* to date have relied on the treatment of the definitive hosts (humans) to remove tapeworm infections (Lightowlers, 2010) and this may have increased the overall susceptibility of the pig population to cysticercosis. Indeed one study found an increase in the prevalence of porcine cysticercosis after mass treatment of the human population with a taeniacide (Keilbach et al., 1989). A major advantage of the incorporation of pig vaccination into control efforts for *T. solium* would be the replacement of naturally acquired immunity in the intermediate host population with vaccine-induced immunity. In this way, the control efforts would not be readily disrupted should new cases of taeniasis be introduced into a control region either through incomplete effectiveness of the control measures or immigration of tapeworm carriers into the control area.

In regions where *T. solium* was highly prevalent, young pigs would be born into a contaminated environment and may be exposed to *T. solium* infection prior to them being vaccinated. It remains unclear whether the TSOL18 has any effect on established cysticerci, notwithstanding the preponderance of non-viable lesions in the vaccinated animals in the field trial described here. For this reason, it would be valuable in a control program to incorporate a measure to ensure that any viable parasites that were already established in animals prior to vaccination were removed as potential sources for transmission of taeniasis to humans. Data are not available about the potential for passive transfer of immunity from dams vaccinated with TSOL antigens to neonates. However, perhaps the simplest method by which pre-natal or neonatal infections with *T. solium* could be eliminated as a potential source of transmission of the parasite could be achieved by inclusion of a single treatment of piglets with oxfendazole at the same time as the animals were vaccinated, as was the case in the trial undertaken in Cameroon (Assana et al., 2010).

## 5 Conclusion

The results obtained by this study reinforce the role of vaccination as a valuable tool to prevent porcine cysticercosis in areas where the disease is endemic, reducing the source of infection in humans and indirectly decreasing the occurrence of neurocysticercosis. With this knowledge, further studies are necessary to assess the feasibility and efficacy of control programs that include vaccination under field conditions.

## Figures and Tables

**Table 1:** Incidence of infection, viable, degenerated and total cyst counts by treatment group in the field vaccination trial, Peru.

Findings	Control	Vaccinated
Assessed by necropsy	107	113
Infected	18	7
Cumulative incidence	16.80%	6.20%
Infected with viable cysts	15	2
Infected with viable muscle cysts	15	1
Incidence of pigs with viable muscle cysts	14.02%	0.88%
Total cysts	34,081^a^	83
Viable cysts	33,416^a^	3
Viable muscle cysts	33,333^a^	1
Degenerated	665	80
Range of cyst counts^b^	1–18,598	1–54
Mean^b^	1893.9	11.9
Median^b^	63.5	6
Degenerated/viable	665/33,416	80/3
Degenerated/viable ratio	0.02^a^	26.67

a Significantly different from the vaccinated group (Wilcoxon signed-rank test *p* < 0.01).

b The range, mean and median were calculated based on the total cysts counts of the infected animals.

**Table 2:** Number of positive and negative pairs of pigs determined by necropsy per treatment group in the field vaccination trial, Peru.

Group		Vaccinated			Total
		Positive	Negative	Unknown	
Control	Positive	4	13	1	18^a^
	Negative	3	80	8	91
	Unknown^b^	0	11	17	28

	Total	7	104	26	137

a Significant difference between the infected control pairs of animals and vaccinated ones (McNemar-chi^2^ test, *p* < 0.05).

b Those animals for which data were available only from 1 member of a pair are shown as individual animals, with the associated pair indicated as unknown. Pairs in which both animals were unavailable for examination are indicated as unknown/unknown.

**Table 3:** Viable and degenerated cyst counts in the pairs of pigs that were infected as determined by necropsy in the field vaccination trial, Peru.

Pair number	Cyst count, TSOL16–TSOL18 group			Cyst count, control group		
	Viable	Degenerated	Total	Viable	Degenerated	Total
12	0	0	0	3271	203	3474
20	1^a^	5	6	0	7	7
23	0	0	0	987	26	1013
25	0	0	0	4	0	4
39	0	0	0	74	1	75
45	0	0	0	1	0	1
46	0	0	0	0	5	5
47^c^	–	–	–	1	0	1
51	0	10	10	0	0	0
56	0	0	0	49	3	52
57	0	0	0	8172	77	8249
73	0	54	54	0	0	0
84	0	0	0	1010	75	1085
86	0	0	0	1	0	1
91	0	1	1	0	200	200
98	0	0	0	18,573	25	18,598
102	0	0	0	1053	31	1084
113	2^b^	5	7	16	11	27
120	0	1	1	36	1	37
122	0	4	4	168	0	168

a The viable cyst was found in the muscle.

b The two viable cysts were found in the brain.

c The vaccinated animal was lost during the experiment and unavailable for necropsy.

**Table 4:** Anatomical distribution of the cysts in the animal carcasses per treatment group in the field vaccination trial, Peru.

Anatomical region	Control			Vaccinated		
	Viable	Degenerated	(%)^a^	Viable	Degenerated	(%)^a^
Right forelimb	4934	105	14.79	0	6	7.23
Left forelimb	4480	62	13.33	0	15	18.07
Right flank	2467	25	7.31	0	1	1.20
Left flank	2585	37	7.69	0	6	7.23
Brain^b^	83	9	0.27	2	0	2.41
Heart	472	24	1.46	0	2	2.41
Cervical-thoracic vertebrae	3713	65	11.09	0	11	13.25
Tongue^b^	525	99	1.83	0	11	13.25
Head muscles^b^	822	61	2.59	0	6	7.23
Right hind limb	4506	60	13.40	0	10	12.05
Left hind limb	4213	86	12.61	1	12	15.66
Sacrum	4616	32	13.64	0	0	0

Total	33,416	665		3	80	

a Proportion of the total cyst count.

b Animals in the control group had a significantly lower odds to have cysts in this anatomical site.
